# Supplementary material for: Sequence tagging for biomedical extractive question answering
Source: Bioinformatics. 2022 Jun 17;38(15):3794–801. doi: 10.1093/bioinformatics/btac397 (PMC9344839; doi:10.1093/bioinformatics/btac397)
Supplement: btac397_Supplementary_Data [file btac397_supplementary_data.pdf]

A Addition Details for Data

A.1 Input Data Format

Figure 1 illustrates a sample of our input data format for sequence tagging configuration. We reformatted the CoNLL-2003 NER task format (Tjong Kim Sang and De Meulder, 2003) to be suitable for a QA task. Each sample consists of a question and its corresponding passage. Samples are separated by blank lines. A question and the unique ID of a sample are located at the first line of a sample which is subsequently followed by the passage. One line represents one word in the passage, and a line consists of a word and its tag. We inserted end-of-sentence (EOS) tokens between sentences.

UNIQUEID	5509f4	What is the method FASP used for?
Proteomic	B	Answer Span
sample	I	
preparation	I	
from	O	
formalin	O	
fixed	O	
and	O	
paraffin	O	
embedded	O	
tissue	O	
.	O	
¶	O	
Preserved	O	

Figure 1: An example of data sample for sequence tagging configuration. A question and a corresponding passage forms a data sample. Paragraph mark (¶) denotes end-of-sentence (EOS) token. In this example, *Proteomic sample preparation* is an answer.

A.2 Original BioASQ dataset

A sample of the original BioASQ dataset (Figure 2) is composed of a question, URLs of PubMed articles (*document*) that are relevant to the given question, and *snippets*, which are manually selected sentences within the given documents. However, the original BioASQ dataset is not directly built for EQA. Consequently, some documents or snippets do not contain answer strings in them. Such documents without answers are excluded when we create training datasets for "Start-end prediction" and "Sequence tagging" configurations.

B Addition Details for Experiments

B.1 Hyperparameters

Baselines We evaluated the baseline models based on their suggestions and did not search for the optimal hyperparameter settings for those models (Yoon et al., 2019; Jeong et al., 2020). However, as one exception, we altered the maximum sequence length (MAX_SEQ_LENGTH) from 384 to 512 for fair comparison with our model. The performances of the baseline models were not significantly affected.

Our models For all of our experiments, we set maximum sequence length to 512 and batch size to 18 (which is the maximum size that could fit onto a TITAN RTX 24GB GPU device). For pre-training our model using the SQuAD v1.1 dataset, we trained the model using a learning rate of 5e-5 for 20 epochs. For fine-tuning our model, we varied the learning rate among 5e-5, 1e-5, and 5e-6 for list and factoid (for Discussion section) question datasets of BioASQ 7b and 8b. We report the performance of models trained with learning rates of 5e-6 for both datasets. The number of maximum training epochs for BioASQ list datasets was 400 epochs, and the batch size was 18. We used checkpoints of training steps at 80,000 steps and 84,000 steps (the last checkpoint before 400 epochs) for the BioASQ 7b and 8b, respectively. Excluding the number of training steps, we did not conduct extensive hyperparameter searching, with each model architecture and dataset combination being validated less than two times. We selected hyperparameter candidates based on the official BioASQ evaluation metrics (F1 for list questions).

B.2 Snippet approach

Table 1: Performance of models on BioASQ 8b List-type questions. Standard deviations are denoted in the parenthesis.

	BioASQ 8b List questions		
System	Prec.	Recall	F1 Score
Jeong et al. (2020) **(Full abstract)**	0.4581 (0.0071)	0.3335 (0.0049)	0.3428 (0.0054)
Jeong et al. (2020) **(Snippet)**	0.5575 (0.0140)	0.4051 (0.0082)	0.4350 (0.0078)
Ours (Seq-Tag) **(Full abstract)**	0.3888 (0.0105)	0.5936 (0.0126)	0.4355 (0.0083)

Table 1 shows the performances of the models with the full abstract settings and the snippet settings. Model of Jeong et al. (2020) performed better in the snippet settings than using the full abstract. As snippets are a more concentrated source of information for answering a question than the full

```
{
        "body": "List Hemolytic Uremic Syndrome Triad.",
        "documents": [
           "http://www.ncbi.nlm.nih.gov/pubmed/26265890",
           "http://www.ncbi.nlm.nih.gov/pubmed/25765799",
           "http://www.ncbi.nlm.nih.gov/pubmed/25011592",
           "http://www.ncbi.nlm.nih.gov/pubmed/24548192",
           "http://www.ncbi.nlm.nih.gov/pubmed/8589282",
           "http://www.ncbi.nlm.nih.gov/pubmed/16387683",
           ...
        ],
        "ideal_answer": [
           "Hemolytic uremic syndrome (HUS) is a clinical syndrome characterized by the triad of anaemia,
         thrombocytopenia, renal failure."
        ],
        "exact_answer": [
           [
              "anaemia"
           ],
           [
              "thrombocytopenia"
           ],
           [
              "renal failure"
           ]
        ],
        "type": "list",
        "id": "56be0da3ef6e394741000007",
        "snippets": [
           {
              "offsetInBeginSection": 0,
              "offsetInEndSection": 155,
              "text": "Hemolytic uremic syndrome (HUS) is a clinical syndrome characterized by the triad
           of thrombotic microangiopathy, thrombocytopenia, and acute kidney injury.",
              "beginSection": "abstract",
              "document": "http://www.ncbi.nlm.nih.gov/pubmed/26265890",
              "endSection": "abstract"
           },
           {
              "offsetInBeginSection": 0,
              "offsetInEndSection": 167,
              "text": "Atypical hemolytic uremic syndrome (aHUS) is a rare disease characterized by the
           triad of microangiopathic hemolytic anemia, thrombocytopenia, and acute renal failure.",
              "beginSection": "abstract",
              "document": "http://www.ncbi.nlm.nih.gov/pubmed/25765799",
              "endSection": "abstract"
           },
           ...,
}
```


Figure 2: An example of BioASQ original dataset for a question (denoted as *body*). URLs of the documents, a list of answers and snippets are highlighted with left parenthesis.

abstract (Figure 2), this phenomenon seems natural. However, as aforementioned in the main script, we do not consider snippets as the passage since the use of snippets may not sufficiently fulfil the final goal of EQA to build an automated process of finding an answer from the given document. Our approach, the sequence tagging approach outperformed Jeong et al. (2020) with the snippet settings, despite the fact that we used full abstract settings.

C Addition Details for the preliminary study

C.1 Annotation scheme

Our question-type classification is based on the type of answer we annotate. The annotation is based on the Google search result including PubMed website. For example, if we look for a question and a number of answer phrases are found, we classify the given question as list type question. On the other hand, if a question neither can be answered with (a) short phrase(s) nor Yes/No, and can be only answered with a sentence, we classify

the question as a "Long answer only" question.

Long answer only If the question requires a sentence-length description as an answer, we annotate it as **Long answer only**, or **Summary** question. Long answer type questions include some questions that require more conditions to be answered with phrases. For example, *What are the guidelines for myringotomy tubes in children?* is a Summary-type question.

Short answer - single phrase If the question requires a short phrase as an answer set, we annotate it as **factoid** question. As an exception, for a question where multiple answer phrases are found but if the phrases indicate the exact same entity, we annotate it as Factoid. As an example, for a question where the answer is "Hydrophilic Interaction Chromatography", and if "Hydrophilic Interaction Chromatography (HILIC)" is in the search result webpage, we consider the question as a factoid question despite the fact that both "Hydrophilic Interaction Chromatography" and "HILIC" can be answer phrases.

Short answer - multiple phrases If the question requires more than one phrase as an answer set, we annotate it as **list** question.

Binary answer If the question is answerable with either Yes or No, we annotate it as **Binary**, or **Yes/No** question. For example, *Is Cozaar an angiotensin receptor blocker?* is annotated as a Yes/No question.

Others If the question is not answerable with current settings. Corresponds to Bad- and "No answer"-questions of NQ. For example *diabetes and neuropath? and sodium and channel and block?* is considered as others-type questions.

C.2 Data Collection

Clinical Questions Collection We collected CQC datasets from NIH [1] and selected IOWA IC QUESTIONS as our source of questions as it is collected from the most diverse set of physicians among the CQC datasets. Among the three types of question strings (i.e., original_question, general_question, and short_question) in the dataset, we choose general_question as our primary source and if the question does not have general_question, we used short_question instead.

[1] https://www.nlm.nih.gov/databases/download/CQC.html

PubMed Queries We collected one-day queries from the NLM FTP site.[2] Following the work of NATURAL QUESTIONS(Kwiatkowski et al., 2019), we applied a simple rule-based query screening algorithm. We selected the queries starting with interrogative words, modal verbs or auxiliary verbs, which are listed as follows (in no particular order): WHEN WHERE WHAT WHO WHOM WHOSE WHICH WHY WHETHER HOW WOULD WILL COULD CAN MAY DOES DO HAVE HAS IS ARE. Queries ending with a question mark are selected as well. Queries shorter than 3 words and longer then 250 characters are excluded. To handle PubMed-specific queries and search operators, we added a few rule-based screening techniques; queries with "WHO" (World Health Organization), "can j " (abbreviation for Canadian Journal), " AND " (wrapped with whitespaces), and " OR " are excluded.

[2] https://ftp.ncbi.nlm.nih.gov/pub/wilbur/DAYSLOG/

References

Minbyul Jeong, Mujeen Sung, Gangwoo Kim, Donghyeon Kim, Wonjin Yoon, Jaehyo Yoo, and Jaewoo Kang. 2020. Transferability of natural language inference to biomedical question answering. *arXiv preprint arXiv:2007.00217*.

Tom Kwiatkowski, Jennimaria Palomaki, Olivia Redfield, Michael Collins, Ankur Parikh, Chris Alberti, Danielle Epstein, Illia Polosukhin, Jacob Devlin, Kenton Lee, et al. 2019. Natural questions: a benchmark for question answering research. *Transactions of the Association for Computational Linguistics*, 7:453–466.

Erik F. Tjong Kim Sang and Fien De Meulder. 2003. Introduction to the CoNLL-2003 shared task: Language-independent named entity recognition. In *Proceedings of the Seventh Conference on Natural Language Learning at HLT-NAACL 2003*, pages 142–147.

Wonjin Yoon, Jinhyuk Lee, Donghyeon Kim, Minbyul Jeong, and Jaewoo Kang. 2019. Pre-trained language model for biomedical question answering. *arXiv preprint arXiv:1909.08229*.
